# RETRACTION: Effects of Exercise in Treating Patients With Venous Leg Ulcers: An Umbrella Review

**DOI:** 10.1111/iwj.70628

**Published:** 2025-04-21

## Abstract

**RETRACTION:** ShenM. , XingM. , JiangH. , ZhangL. , ChenC. , MaY. , and MaY. , “Effects of Exercise in Treating Patients With Venous Leg Ulcers: An Umbrella Review,” International Wound Journal 21, no. 2 (2024): e14645, 10.1111/iwj.14645.PMC1083091740261158

The above article, published online on 31 January 2024, in Wiley Online Library (http://onlinelibrary.wiley.com/), has been retracted by agreement between the journal Editor in Chief, Professor Keith Harding; and John Wiley & Sons Ltd. Following an investigation by the publisher, all parties have concluded that this article was accepted solely on the basis of a compromised peer review process. In addition, further investigation by the publisher found that the methodology, data extraction and quality assessment were inadequately described, and that there are inconsistencies between the search strategy defined in the methods and the presented data. The editors have therefore decided to retract the article. The authors did not respond to our notice regarding the retraction.

**RETRACTION:** M. Shen , M. Xing , H. Jiang , L. Zhang , C. Chen , Y. Ma , and Y. Ma , “Effects of Exercise in Treating Patients With Venous Leg Ulcers: An Umbrella Review,” International Wound Journal 21, no. 2 (2024): e14645, 10.1111/iwj.14645.PMC1083091740261158

The above article, published online on 31 January 2024, in Wiley Online Library (http://onlinelibrary.wiley.com/), has been retracted by agreement between the journal Editor in Chief, Professor Keith Harding; and John Wiley & Sons Ltd. Following an investigation by the publisher, all parties have concluded that this article was accepted solely on the basis of a compromised peer review process. In addition, further investigation by the publisher found that the methodology, data extraction and quality assessment were inadequately described, and that there are inconsistencies between the search strategy defined in the methods and the presented data. The editors have therefore decided to retract the article. The authors did not respond to our notice regarding the retraction.

